# Gut dysbiosis and inflammatory blood markers precede HIV with limited changes after early seroconversion

**DOI:** 10.1016/j.ebiom.2022.104286

**Published:** 2022-09-27

**Authors:** Jennifer A. Fulcher, Fan Li, Nicole H. Tobin, Sara Zabih, Julie Elliott, Jesse L. Clark, Richard D'Aquila, Brian Mustanski, Michele D. Kipke, Steven Shoptaw, Pamina M. Gorbach, Grace M. Aldrovandi

**Affiliations:** a Division of Infectious Diseases, Department of Medicine, David Geffen School of Medicine at UCLA, Los Angeles, CA 90095, USA; b VA Greater Los Angeles Healthcare System, Los Angeles, CA 90073, USA; c Division of Infectious Diseases, Department of Pediatrics, David Geffen School of Medicine at UCLA, Los Angeles, CA 90095, USA; d Division of Infectious Diseases, Northwestern University Feinberg School of Medicine, Chicago, IL 60611, USA; e Department of Medical Social Sciences, Northwestern University Feinberg School of Medicine, Chicago, IL 60611, USA; f Institute for Sexual and Gender Minority Health and Wellbeing, Northwestern University, Chicago, IL 60611, USA; g Children's Hospital Los Angeles, Los Angeles, CA 90027, USA; h Department of Pediatrics, Keck School of Medicine at the University of Southern California, Los Angeles, CA 90027, USA; i Department of Family Medicine, David Geffen School of Medicine at UCLA, Los Angeles, CA 90095, USA; j Department of Epidemiology, Fielding School of Public Health, University of California Los Angeles, Los Angeles, CA 90095, USA

**Keywords:** HIV, Microbiome, HIV acquisition, Cytokines

## Abstract

**Background:**

Alterations in the gut microbiome have been associated with HIV infection, but the relative impact of HIV versus other factors on the gut microbiome has been difficult to determine in cross-sectional studies.

**Methods:**

To address this, we examined the gut microbiome, serum metabolome, and cytokines longitudinally within 27 individuals before and during acute HIV using samples collected from several ongoing cohort studies. Matched control participants (*n*=28) from the same cohort studies without HIV but at similar behavioral risk were used for comparison.

**Findings:**

We identified few changes in the microbiome during acute HIV infection, but did find alterations in serum metabolites involving secondary bile acid (lithocholate sulfate, glycocholenate sulfate) and amino acid metabolism (3-methyl-2-oxovalerate, serine, cysteine, N-acetylputrescine). Greater microbiome differences, including decreased *Bacteroides spp* and increased *Megasphaera elsdenii*, were seen when comparing pre-HIV infection visits to matched at-risk controls. Those who acquired HIV also had elevated inflammatory cytokines (TNF-α, B cell activating factor, IL-8) and bioactive lipids (palmitoyl-sphingosine-phosphoethanolamide and glycerophosphoinositol) prior to HIV acquisition compared to matched controls.

**Interpretation:**

Longitudinal sampling identified pre-existing microbiome differences in participants with acute HIV compared to matched control participants observed over the same period. These data highlight the importance of increasing understanding of the role of the microbiome in HIV susceptibility.

**Funding:**

This work was supported by NIH/NIAID (K08AI124979; P30AI117943), NIH/NIDA (U01DA036267; U01DA036939; U01DA036926; U24DA044554), and NIH/NIMH (P30MH058107; R34MH105272).

**Research in context**

**Evidence before this study**

Changes in the gut microbiome have been associated with HIV infection in numerous studies. However, many prior studies were limited by cross-sectional design and varying degrees of comparability between the HIV and control groups in regards to potential confounding factors. More recent studies showed that similar microbiome changes can be associated with sexual behavior, independent of HIV status, making the relationship between HIV and the microbiome less clear. Thus, the precise effects of HIV on the gut microbiome remain in question.

**Added value of this study**

This study takes advantage of valuable longitudinal samples spanning the period before and during acute HIV acquisition to examine changes in the gut microbiome and serum metabolites/cytokines occurring with early HIV infection. Importantly, this study also includes well-matched control samples without HIV collected over the same time. We found minimal microbiome changes during acute HIV acquisition; however, pre-existing microbiome differences were identified between those who later acquired HIV and control samples without HIV. Serum metabolic changes associated with secondary bile acid and amino acid metabolism were observed with early HIV seroconversion.

**Implications of all the available evidence**

Gut microbiome changes, including decreased *Bacteroides*, are associated with HIV in multiple studies. However, results from this study showed that these microbiome differences may in fact be pre-existing as few changes were observed in the gut microbiome during acute HIV acquisition. This may suggest that these microbiome changes could play a role in susceptibility to HIV infection. This study cannot exclude microbiome changes occurring during chronic HIV, and this remains an important area for ongoing study. Metabolic changes with early HIV seroconversion include secondary bile acid and amino acid metabolism, and may involve interactions with gut bacteria.

Alt-text: Unlabelled box

## Introduction

The gastrointestinal tract is a primary site of HIV transmission and early replication, and alterations in this mucosal environment contribute to disease progression.^1^, ^2^, ^3^, ^4^ Ongoing mucosal inflammation during HIV infection leads to microbial dysbiosis and translocation,^5^, ^6^, ^7^, ^8^ and indicators of microbial translocation have been repeatedly associated with systemic inflammation in clinical studies.^5^^,^^8^, ^9^, ^10^, ^11^, ^12^ However, the time course and drivers of microbial dysbiosis in HIV are not fully understood.

Changes in the gut microbiome composition, or dysbiosis, have been associated with HIV in numerous studies.^8^^,^^9^^,^^11^^,^^13^, ^14^, ^15^ Most common reported findings include increases in potentially pathogenic Proteobacteria, increases in inflammatory genus *Prevotella*, and decreased commensals such as Bacteroidetes and Firmicutes.^8^^,^^9^^,^^11^^,^^13^ Despite the multitude of quality studies, the generalizability of microbiome data has been limited by differences in sample collection, cohorts, and unknown influence of multiple confounders.

Many factors may contribute to microbiome composition including age, geography, diet, antibiotics, and behaviors.^16^^,^^17^ Recent studies have shown that men who have sex with men (MSM) have a characteristic microbiome composition different than men who have sex with women, and some of these microbiome changes are similar to those attributed to HIV in other cross-sectional studies.^18^^,^^19^ To date, few studies have longitudinally examined microbiome composition in individuals before and after HIV acquisition, likely due to the rarity of such samples. A recent study from Chen et al examined fecal samples from MSM in the Multicenter AIDS Cohort Study (MACS) and found that HIV-1 seroconverters had distinct microbiome changes prior to HIV-1 infection including decreased Bacteroidaceae, and these changes associated with increased plasma inflammatory biomarkers. After seroconversion, increases in Prevotellaceae and Victivallaceae were associated with faster disease progression.^20^ This highlights the need for further examination of the microbiome prior to HIV acquisition and in early infection to better understand its role in acquisition risk and disease trajectory.

Microbiome alterations in HIV have been linked to changes in host metabolites and immune biomarkers. Alterations in plasma metabolites related to bile acid metabolism and amino acid metabolism have been associated with HIV,^21^ and metabolic changes have been further associated with disease progression in multiple studies.^21^, ^22^, ^23^ Serrano-Villar et al showed that HIV also resulted in specific changes in gut bacterial metabolites related to amino acid metabolism compared to other inflammatory diseases.^24^ The timing of these metabolic changes, and how they may relate to HIV acquisition, is not known.

In this study we sought to comprehensively examine the longitudinal microbiome, metabolic, and immune biomarker changes within individuals before and after early HIV seroconversion, as compared to a control cohort of individuals with similar demographic and behavioral characteristics who did not acquire HIV. We hypothesized that early changes in the microbiome and associated metabolites following HIV infection may help identify drivers and mechanisms leading to HIV-associated dysbiosis and immune activation. We further hypothesized that pre-existing differences in microbiome composition and/or metabolite production in those who acquired HIV compared to behaviorally-matched control persons may contribute to HIV mucosal transmission risk.

## Methods

### Study groups and sample collection

#### Participating cohort studies

This study used archived samples from three ongoing cohort studies and one completed study. All studies were evaluated and approved by the Institutional Review Boards of the respective institutions (UCLA, CHLA, Northwestern University). All participants provided signed informed consent for participation in these studies. The mSTUDY is a longitudinal cohort of racial/ethnic minority men who have sex with men half actively using drugs based on Los Angeles, CA. Nine participants from mSTUDY met criteria and were included in the HIV cases group (32 visits) and 20 participants in the control group (40 visits). mSTUDY samples used in this study were collected between 2014 and 2018. The Healthy Young Men's Cohort Study (HYM) is a longitudinal cohort of diverse young men who have sex with men based in Los Angeles, CA. Seven participants from HYM were included in the HIV cases group (14 visits). HYM samples used in this study were collected between 2016 and 2018. RADAR is a longitudinal cohort of young men who have sex with men based in Chicago, IL.^25^ Three participants from RADAR met criteria and were included in the HIV cases group (6 visits). RADAR samples used in this study were collected between 2015 and 2017. The Rectal STI study was designed to assess the association between incident rectal gonococcal and/or chlamydial infection and HIV acquisition among MSM in Lima, Peru and was conducted in 2017. Eight participants from this study met criteria and were included in the HIV cases group (23 visits) and 8 participants in the control group (24 visits).

#### Study design

The ‘HIV cases’ group included all participants with available rectal specimens from both before and after positive HIV test. The pre-HIV samples were collected at study visits with documented negative HIV test (either rapid HIV test or 4^th^ generation Ag/Ab ELISA), and the post-HIV samples were collected at study visits with documented positive HIV test (4^th^ generation Ag/Ab ELISA). Date of first positive HIV test was obtained from cohort study records. Data on antiretroviral therapy (ART) was obtained from cohort study records as available. Of the 41 post-HIV samples, 15 were collected prior to ART, 18 were collected on ART, and 8 samples had unknown ART status. A matched ‘Control’ group was created to include samples collected over the same time period from participants who did not acquire HIV. This group included samples from mSTUDY and Peru, and was created using frequency matching on the following covariates: age, race/ethnicity, visits with self-reported drug use (by drug type), number of receptive anal intercourse partners within the previous 3 or 6 months, and rectal sexually transmitted infection prevalence. Statistical summaries of these variables by group are shown in Table 1.

**Table 1:** Demographics of study participants separated by country.

		United States			Peru		
		HIV Cases	Control	*p*-value^a^	HIV Cases	Control	*p*-value^a^
Total participants		19	20		8	8	
Total visits		52	40		23	24	
Duration of follow-up (months)							
Total	Mean (SD)	13 (5.4)	11 (4.1)	0.13	5.6 (1.1)	6 (0)	>0.99
	Median (IQR)	13 (10–18)	11 (7–12)		6 (6–6)	6 (6–6)	
Prior to HIV	Mean (SD)	7.7 (4.1)	-		3 (1.6)	-	-
	Median (IQR)	8 (6–9)	-		3 (3–3)	-	
After HIV	Mean (SD)	6.4 (4.7)	-		2.6 (1.1)	-	-
	Median (IQR)	6 (3–9.5)	-		3 (3–3)	-	
							
Age (years)	Mean (SD)	25 (6.5)	25 (2.6)	0.06	27 (6.4)	28 (7)	0.74
	Median (IQR)	23 (21–26)	25 (23–27)		27 (21–32)	27 (23–31)	
Race/Ethnicity	Number (%)			0.71			>0.99
Hispanic/Latino		9 (47.4%)	9 (45%)		8 (100%)	8 (100%)	
Black		8 (42.1%)	9 (45%)		0	0	
White		1 (5.3%)	2 (10%)		0	0	
Other		1 (5.3%)	0		0	0	
Body Mass Index (BMI)	Mean (SD)	22 (3.6)	27 (5.9)	0.06	NA	NA	-
	Median (IQR)	22 (20–24)	26 (23–31)				
Receptive anal intercourse partners	Mean (SD)	5.2 (8.2)	3.7 (3.9)	0.47	4.3 (3.1)	2.8 (1.9)	0.13
	Median (IQR)	3 (1–6)	3 (1–5.8)		3 (2.8 –5.3)	2 (1–4)	
Visits with rectal *N. gonorrhoeae*	Number (%)	6 (11.5%)	2 (5%)	0.46	2 (8.7%)	2 (8.3%)	>0.99
Visits with rectal *C. trachomatis*	Number (%)	5 (9.6%)	3 (7.5%)	>0.99	5 (21.7%)	4 (16.7%)	0.72
Post-HIV visits prior to antiretroviral therapy (ART)^e^	Number (%)	7 (26%)	-	-	8 (57%)	-	-
Drug use (self-report)^b^	Number (%)						
Visits with cocaine		17 (32.7%)	6 (15%)	0.06	2 (8.7%)	0	0.23
Visits with methamphetamine		19 (36.5%)	14 (35%)	>0.99	1 (4.4%)	0	0.49
Visits with ecstasy		15 (28.8%)	7 (17.5%)	0·23	NA	NA	-
Visits with cannabis		38 (73.1%)	21 (52.5%)	0.05	3 (13%)	2 (8.3%)	0.67
Visits with poppers		22 (40.4%)	10 (25%)	0.18	2 (8.7%)	0	0.23
Visits with party drugs^c^		8 (15.4%)	3 (7.5%)	0.34	NA	NA	-
Visits with prescription drugs^d^		6 (11.5%)	5 (12.5%)	>0.99	NA	NA	-
Visits with no drugs		5 (9.6%)	6 (15%)	0.52	17 (74%)	22 (91.7%)	0.14

SD, standard deviation; NA, data not available.

a *p*-values calculated using Chi squared, Fishers exact, Mann-Whitney, or unpaired t-tests as appropriate.

b self-report of drug use over past 6 months for United States cohorts or over past 3 months for Peru cohort.

c party drugs included GHB, ketamine, mushrooms, LSD.

d prescription drugs included benzodiazepines, prescription opiates.

e total visits includes 2 visits (United States) and 6 visits (Peru) with unknown ART status.

#### Demographic and behavioral data

All demographic and behavioral data was obtained from cohort study records. mSTUDY, HYM, and RADAR conduct visits every 6 months with collection of clinical data including laboratories, urine drug screen, sexually transmitted infection (STI) screen, biospecimens, and computer assisted self-interview detailing sexual behavior and substance use. The Peru study conducted visits every 3 months with collection of clinical data, STI screen, and biospecimens. Self-reported drug use was based on past 6 months use (except for Peru study which was 3 months use due to study schedule).

#### Rectal samples

For mSTUDY, HYM, and RADAR rectal swabs were self-collected by participants then frozen at −80 °C until shipping and processing in bulk. For Peru, rectal sponges (Merocel) were collected via anoscopy under direct visualization and mucosal contact for two minutes then at −80 °C until shipping and processing in bulk.

#### Blood samples

Blood samples were only available from a subset of participants so serum analyses (metabolomics, cytokine quantification) were performed using archived serum specimens from *n*=9 HIV case and *n*=20 control participants. All blood samples were from the mSTUDY cohort (Los Angeles).

### Shotgun metagenomic sequencing: DNA extraction, library preparation, and sequencing

Swab samples were extracted using bead-beating with Lysing Matrix E beads (MP Biomedicals, Burlingame, CA) in RLT-Plus lysis buffer from the AllPrep DNA/RNA isolation kit (Qiagen). DNA extraction was completed using the AllPrep DNA/RNA isolation kit per manufacturer's protocol. Sponge samples were first eluted as previously described,^26^ then extraction was performed as described above for swabs. Barcoded libraries were prepared using the Qiagen FX DNA Library Kit following manufacturer's protocols and sequenced using Illumina NextSeq500 2×150bp v2 chemistry to a target depth of 10 million read pairs per sample. Positive controls comprising our bacterial mock community and negative controls were also sequenced to a lower target depth of 500,000 reads per sample. Reads from all samples including controls were preprocessed and quality filtered using Trim Galore (available at https://github.com/FelixKrueger/TrimGalore). Host-derived reads were removed using KneadData (available at https://bitbucket.org/biobakery/kneaddata). Taxonomic classification and quantification at the species level was performed using kraken 2 and Bracken.^27^^,^^28^ Gene family abundance, pathway abundance, and pathway coverage was calculated using HUMAnN2.^29^

### Microbiome analysis

Bacterial abundances were summarized at the species level and additional analyses performed using the ‘phyloseq’ and ‘vegan’ R packages. Alpha diversity was calculated using the Chao1 index, and principal coordinates analysis (PCoA) and permutational multivariate analysis of variance (PERMANOVA) were performed using weighted UniFrac distances. A post hoc power analysis based on simulation studies using the ‘micropower’ R package with *n*=28 subjects per group was performed showing 90% power to detect a PERMANOVA effect size of ω^2^=<0.005 at *α*=0.05.^30^ These estimates are in line with empirical power calculations suggesting we are sufficiently powered to detect both intra- and inter-individual differences.^31^ To examine taxa associated with HIV seroconversion we used mixed effects modeling with the ‘MaAsLin2’ package in R.^32^ Absolute taxa abundances were normalized (TSS) in the ‘MaAsLin2’ package. Group (CaseNegative, CasePositive, ControlNegative) was used as a fixed effect and subject (for repeated sampling) was used as a random effect. Not all participants were started on ART prior to the first post-HIV sample, thus in the case-negative vs case-positive (seroconversion) comparison “OnART” was used as an additional fixed effect. A sensitivity analysis excluding all samples on ART was also performed with no differences in the significant results. *P*-values were corrected for multiple comparisons using false discovery rate (FDR). Random forests classification models were constructed separately for each data type and comparison using the ‘ranger’ R package (v0.13.1). Species-level relative abundances were used as input. Tenfold cross-validation was used to identify the optimal number of features in each model up to a maximum of fifty features to aid interpretability. One hundred forests each comprising 10,000 trees were used to obtain mean feature importance values. A sparse model was then constructed for each comparison and data type containing the selected number of features with the highest importance (calculated as mean decrease in accuracy). The sparse model was then trained on the entire dataset with accuracy calculated from this sparse model.

### Serum cytokine quantification

Serum cytokines/biomarkers were measured using custom Luminex multiplex bead arrays and ELISAs (R&D Systems) per manufacturer's instructions. All samples were run in duplicate and processed in bulk to reduce inter-assay variability. Samples with a mean coefficient of variation (%CV) >20% were repeated. Values below the lower limit of quantification were imputed as LLOQ/2.

### Serum metabolomics

Untargeted global metabolomics analysis was performed on serum samples by Metabolon Inc (Morrisville, NC) using ultra-high-performance liquid chromatography tandem mass spectrometry. Biochemical identification was performed using Metabolon's chemical library consisting of over 3300 compounds using three criteria: retention index (RI) within a narrow RI window of the proposed identification, accurate mass match to the library +/− 10 ppm, and the MS/MS forward and reverse scores between the experimental data and authentic standards. Data were normalized and peaks quantified using area-under-the-curve.

### Metabolomics analysis

Log-transformed normalized data was used for mixed effects modeling using ‘MaAsLin2’ as described above for microbiome. Normalization was set to “none” and group (CaseNegative, CasePositive, ControlNegative) was used as a fixed effect with subject (for repeated sampling) used as a random effect. In the case-negative vs case-positive (seroconversion) comparison, “OnART” was used as an additional fixed effect. *P*-values were corrected for multiple comparisons using false discovery rate (FDR). Random forests classification models were constructed as described above using the ‘ranger’ R package (v0.13.1). Standardized metabolite abundances were used as the input.

### Multi-omics analysis

Hierarchical all-versus-all association (HAllA, available at https://github.com/biobakery/halla) was used to identify significant associations between microbial species-level relative abundances and metabolite levels. Integrative analysis of metagenomic, metabolomic, and cytokine quantifications was performed using the DIABLO framework from the ‘mixOmics’ R package (v6.17.27). Species-level bacterial relative abundances, HUMANn2-computed relative pathway abundances, standardized metabolite abundances, and standardized cytokine levels were used as input. The block link within the design matrix was set at 0.1, and tenfold cross-validation was used to tune the number of components and the number of features in the final model as described here: http://mixomics.org/mixdiablo/diablo-tcga-case-study/.

### Statistical analysis

Statistics pertaining to microbiome, metabolomics, and multi-omics analyses are described above and performed in R version 4.1.2. All other analysis were performed using Prism 9 (v9.3.1). Cytokines/biomarkers were compared using Kruskal-Wallis tests and Wilcoxon signed rank test for paired before and after HIV samples. Longitudinal data curves were estimated using smoothing splines.

### Role of the funding source

The funding sources had no role in the study design, data collection, data analysis, or writing/publishing the report.

## Results

### Study participants

Archived samples were selected from three ongoing cohort studies in the United States (mSTUDY, HYM, RADAR) and one completed study in Peru. The ‘HIV cases’ group acquired HIV during the observation period, and the ‘Control’ group did not acquire HIV (see Methods for details). The average age of study participants was 25.9 years, included predominantly racial/ethnic minorities (61% Latino and 31% Black), and 74% persons who use drugs (Table 1). All participants were assigned male at birth. The average total observation period was 10 months (range 4 to 24 months), with an average of 3 visits per participant (range of 2 to 5). There were no significant differences in clinical and demographic characteristics between the HIV cases and control groups. Importantly, the HIV cases and control groups were also matched in terms of behavioral factors that affect HIV risk (Table 1).

### Limited changes occur within individuals during early HIV-1 infection

**Figure 1:**
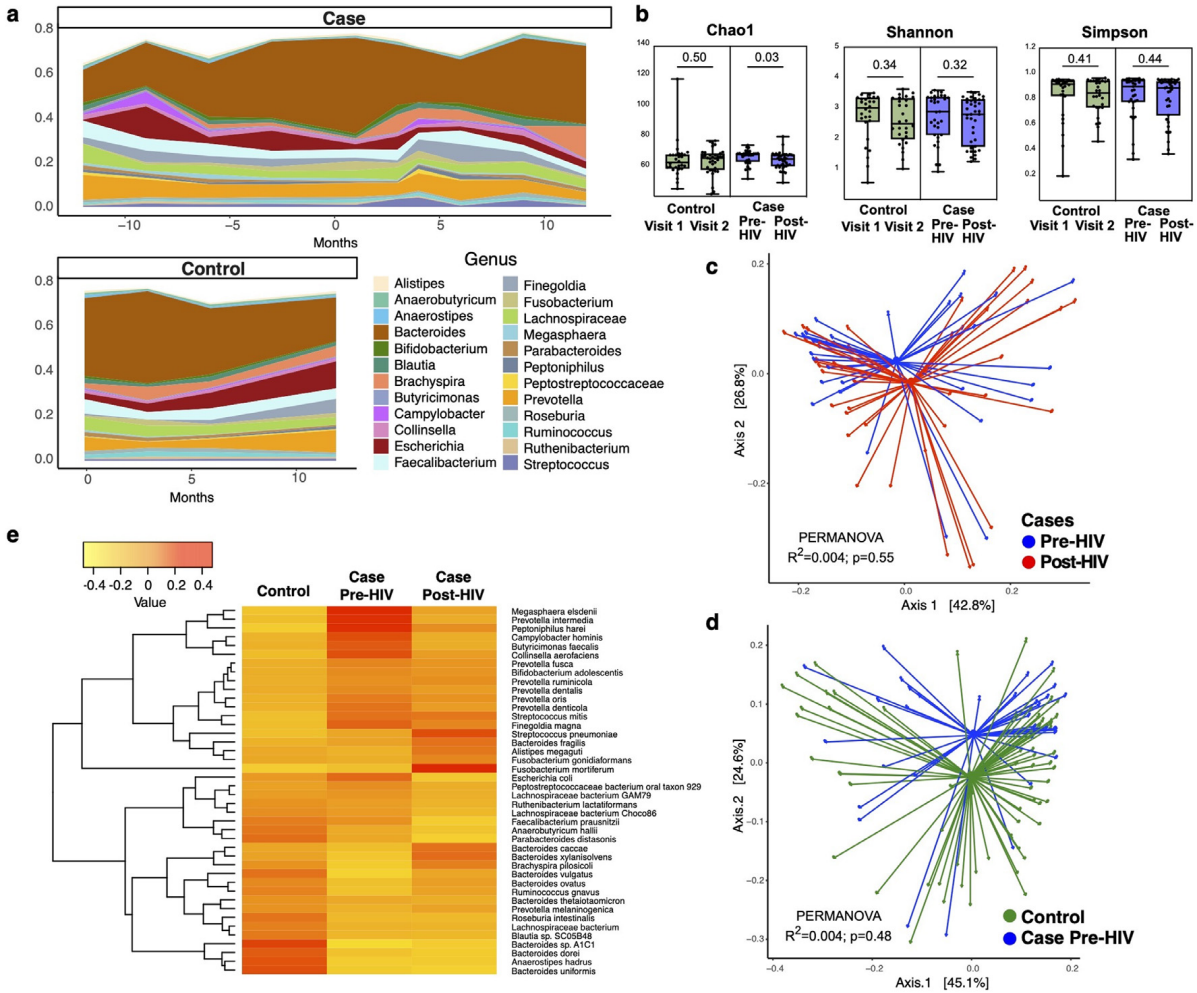
**Gut microbiome comparisons between HIV cases and control persons. (a)** Microbiome composition shown as relative abundance by genus over time in HIV cases (top) and control persons (bottom). Months are relative to positive HIV test which is at time=0 months for HIV cases. **(b)** Comparisons in alpha diversity between control visits and HIV cases pre-HIV and post-HIV. Box line denotes median and whiskers min to max values. *p*-values determined by Wilcoxon signed rank tests. **(c)** Principal coordinates analysis of HIV cases group before HIV (blue) and after HIV (red). **(d)** Principal coordinates analysis of HIV cases pre-HIV samples (blue) and control group (green). **(e)** Heatmap showing relative abundances between control, pre-HIV, and post-HIV samples for all bacterial species with at least 1% relative abundance in at least 10% of all study samples.(For interpretation of the references to color in this figure legend, the reader is referred to the web version of this article.)

Changes in the gut microbiome before and after early HIV seroconversion were examined using shotgun metagenomic sequencing from longitudinal rectal swab samples. Relative microbiome composition changes by genus over time are shown in Figure 1A. Bacterial diversity (Chao1) was found to be decreased during acute HIV within the HIV cases group and no other significant changes in alpha diversity were observed (Figure 1B, Supplementary Table 1). The most significant drivers of microbiome variation were race/ethnicity (PERMANOVA *p*=0.003) and geographic cohort (*p*=0.001); HIV serostatus was not a significant contributor to microbiome variation (*p*=0.610). This is further evidenced in the principal coordinates analysis showing limited differences in the HIV cases group between pre- and post-HIV samples (Figure 1C). In contrast, we did observe differences when comparing pre-HIV acquisition cases samples to the control group who did not acquire HIV (Figure 1D), suggesting that there may be pre-existing microbiome differences in those who acquire HIV. The heatmap in Figure 1E further depicts the clear differences between the pre-HIV case and control groups, with much less difference seen between the pre- and post-HIV samples.

**Figure 2:**
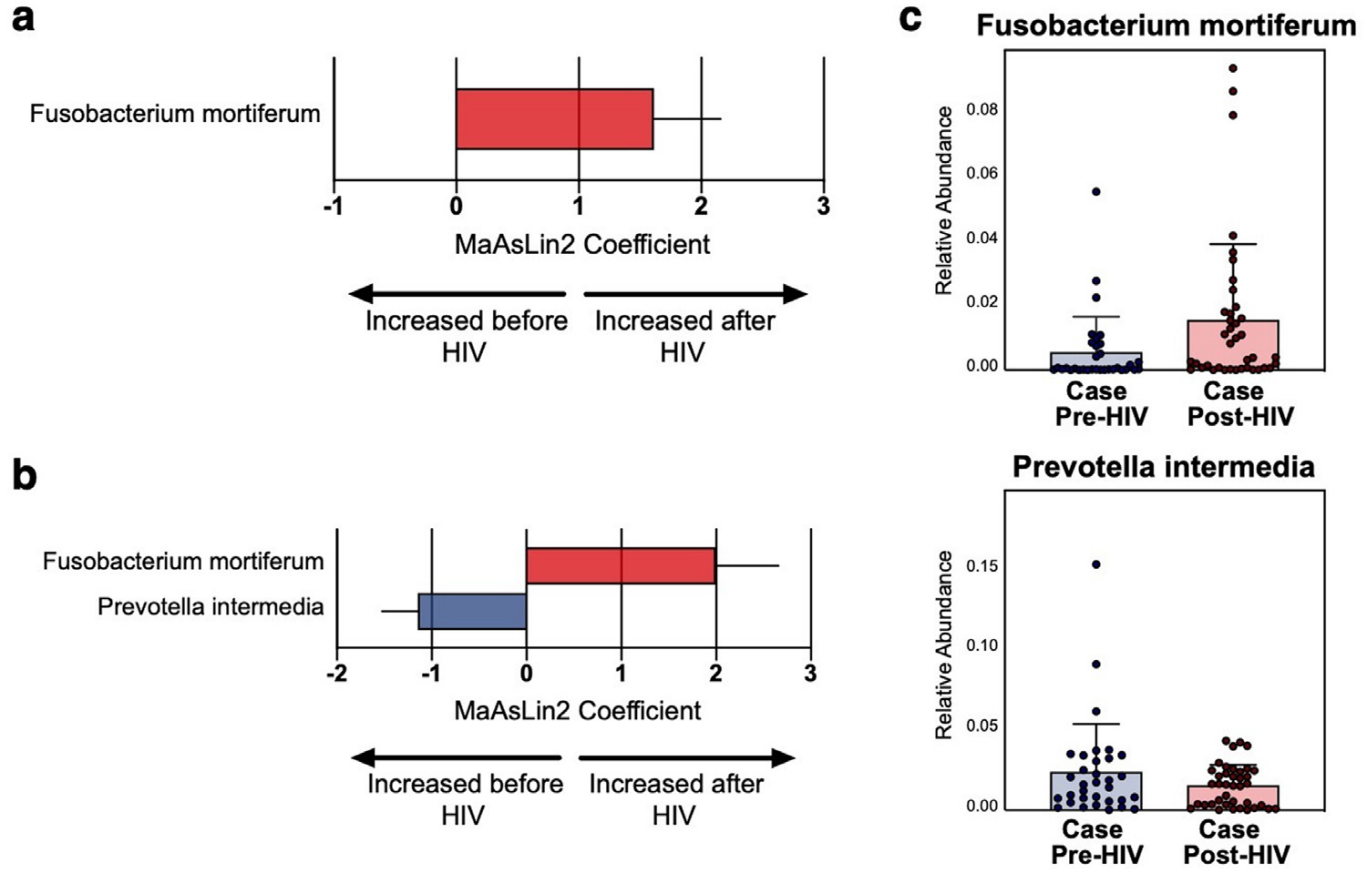
**Changes in gut bacteria before and after early HIV seroconversion**. Bacteria were identified by linear mixed effects models comparing pre-HIV and post-HIV samples in the HIV cases group. Only those with adjusted *p*-value (q) <0.25 are shown and error bars represent standard error. **(a)** shows the full study population and **(b)** shows the United States cohorts only. **(c)** Relative abundance of significant bacteria pre- and post-HIV. Boxes denote mean and error bars represent standard deviation.

We used generalized linear mixed models to identify species that distinguish the microbiome before and during acute HIV (Supplementary Table 2). When examining longitudinal samples from before and after early HIV seroconversion, only increased *Fusobacterium mortiferum* differentiated the post-HIV microbiome (Figure 2A). The same feature was identified by a predictive modeling approach using random forests. Given the significant contribution of geography in shaping the microbiome,^16^ we performed subgroup analyses using only cohorts from the United States. Small sample size prohibited similar subgroup analyses using only Peru samples. When examining the United States subgroup, increased *Fusobacterium mortiferum* and decreased *Prevotella intermedia* were discriminatory features of the post-HIV microbiome (Figure 2B). Relative abundance of these key bacterial species is shown in Figure 2C. To account for the possible contribution of antiretroviral therapy (ART) in the post-HIV samples, analyses were performed using ART as a fixed effect. Other confounding factors were balanced in the groups by design and not included as additional covariates.

### Pre-existing microbiome differences distinguish HIV cases from the control group

**Figure 3:**
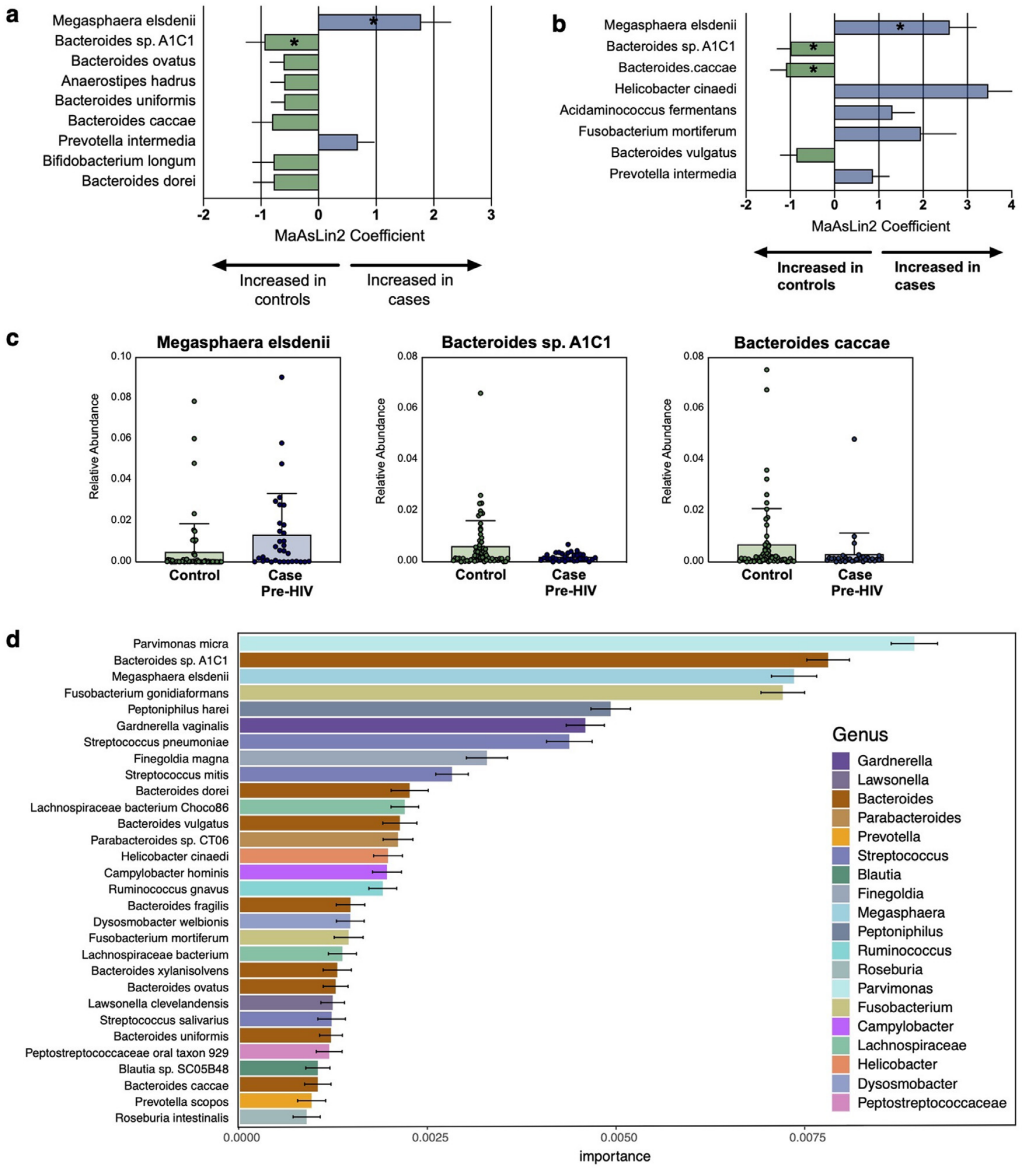
**Bacteria that distinguish control persons and HIV cases**. Bacteria were identified by linear mixed effects models comparing pre-HIV cases with control group samples. Only those with adjusted *p*-value (q) <0.25 are shown and * indicates those with q<0.1. Error bars represent standard error. **(a)** shows the full study population and **(b)** shows the United States cohorts only. **(c)** Relative abundance of significant bacteria between pre-HIV cases and control group. Boxes denote mean and error bars represent standard deviation. **(d)** Bacteria features identified by random forests that distinguish pre-HIV cases from controls ordered by relative importance in the model. Colors indicate bacterial genus, error bars represent standard deviation.(For interpretation of the references to color in this figure legend, the reader is referred to the web version of this article.)

To examine potential features that may identify those that eventually acquired HIV from those that did not, we compared the pre-HIV case samples and the control group samples using generalized linear mixed effects models (Figure 3A, Supplementary Table 2). Again, subgroup analyses using only cohorts from the United States were performed (Figure 3B). Many more discriminatory features were identified between the pre-HIV case and control groups, including increased abundance of multiple *Bacteroides* species in the control group. Increased *Megasphaera elsdenii, Acidaminococcus fermentans,* and *Helicobacter cinaedi* characterized the pre-HIV microbiome in those who later acquired HIV. Two of the species that differed before and after early HIV seroconversion, *Fusobacterium mortiferum* and *Prevotella intermedia* (Figure 2B), were also selected in this comparison again suggesting that some microbiome differences associated with HIV may in fact be pre-existing. Relative abundance of the most significant discriminatory bacterial species is shown in Figure 3C. Random forests modeling trained on the entire dataset identified similar predictive features and correctly classified 74.2% of the pre-HIV case samples from controls (Figure 3D, Supplementary Table 3).

### Early HIV seroconversion induces changes in serum metabolites involving bile acid and amino acid metabolism

**Figure 4:**
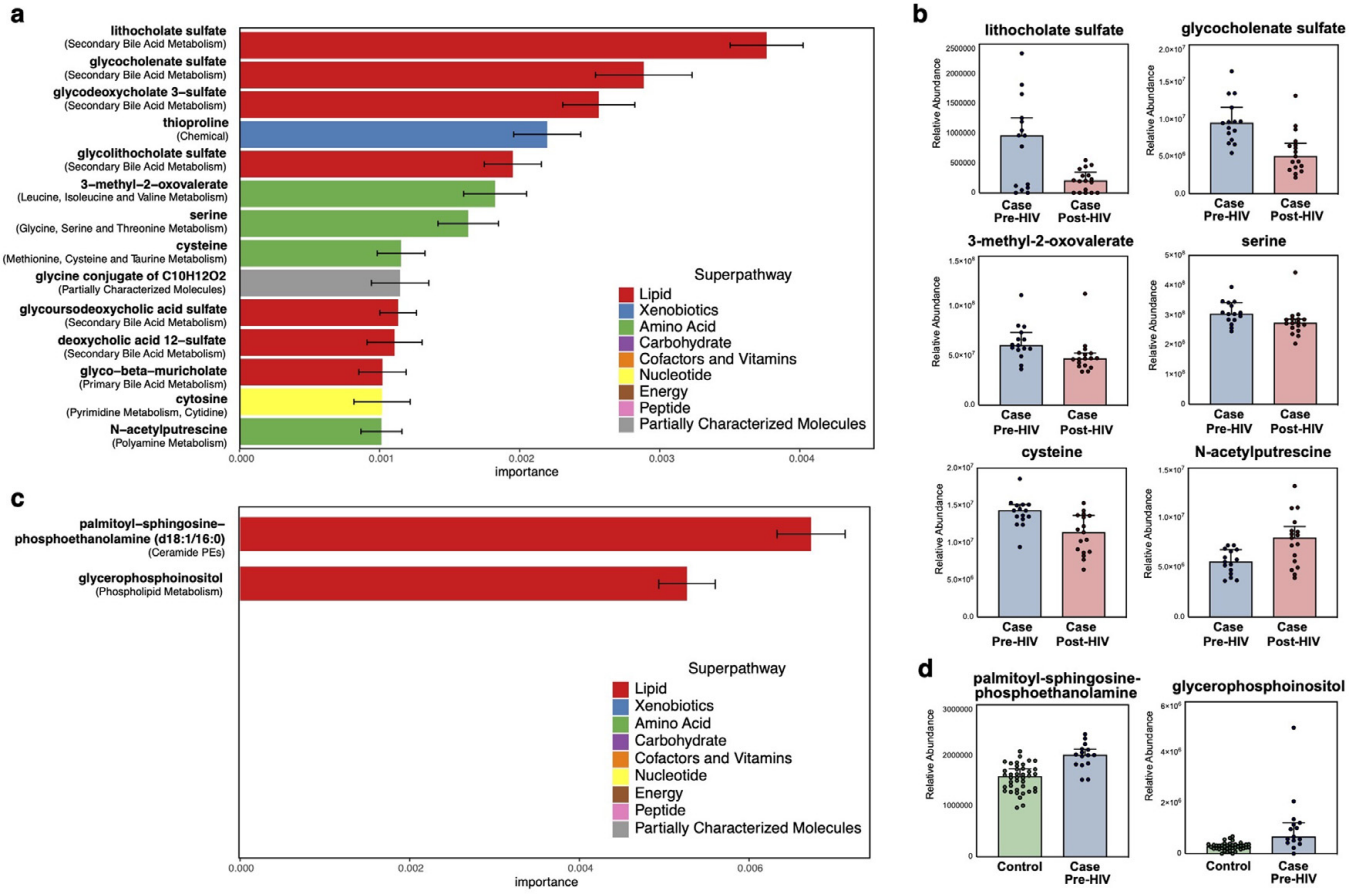
**Metabolites that differ with HIV acquisition and seroconversion. (a)** Metabolite features identified by random forests that distinguish pre-HIV and post-HIV samples in the HIV cases group ordered by relative importance in the model. Colors indicate metabolic superpathway, error bars represent standard deviation. **(b)** Relative abundance of metabolites before and after HIV in HIV cases group. Boxes denote mean and error bars represent standard deviation. **(c)** Metabolite features identified by random forests that distinguish pre-HIV cases from controls ordered by relative importance in the model. Colors indicate metabolic superpathway, error bars represent standard deviation. **(d)** Relative abundance of metabolites between pre-HIV cases and control group. Boxes denote mean and error bars represent standard deviation.(For interpretation of the references to color in this figure legend, the reader is referred to the web version of this article.)

Untargeted metabolomics analysis was performed on paired serum samples from a subset of the study population where blood specimens were available, and included *n*=9 participants in the HIV cases group (total of 32 time points) and *n*=20 participants in the control group (total of 40 time points). All blood samples were from the same cohort in the United States (mSTUDY). No significant metabolites were identified when comparing plasma metabolic profiles before and after early HIV seroconversion using linear mixed effects models, possibly due to sample size limitations. However, random forests predictive modeling using the entire dataset was able to distinguish samples before and after early HIV seroconversion with 84.38% accuracy, and identified key metabolic features that distinguish pre- and post-HIV seroconversion (Figure 4A, Supplementary Table 4). These include secondary bile acids and their derivatives (lithocholate sulfate, glycocholenate sulfate) and amino acid metabolism components (3-methyl-2-oxovalerate, serine, cysteine, N-acetylputrescine) (Figure 4B).

We next compared serum metabolites from the pre-HIV case sample to controls to identify metabolites that may be involved in HIV acquisition risk. Again, no significant metabolites were identified using linear mixed effects models. Random forests modeling trained on the entire dataset achieved an accuracy of 87.27% and identified two bioactive lipids (palmitoyl-sphingosine-phosphoethanolamide and glycerophosphoinositol) that distinguished HIV cases from controls (Figure 4C and 4D).

### HIV cases showed elevated serum inflammatory cytokines prior to HIV-1 acquisition

**Figure 5:**
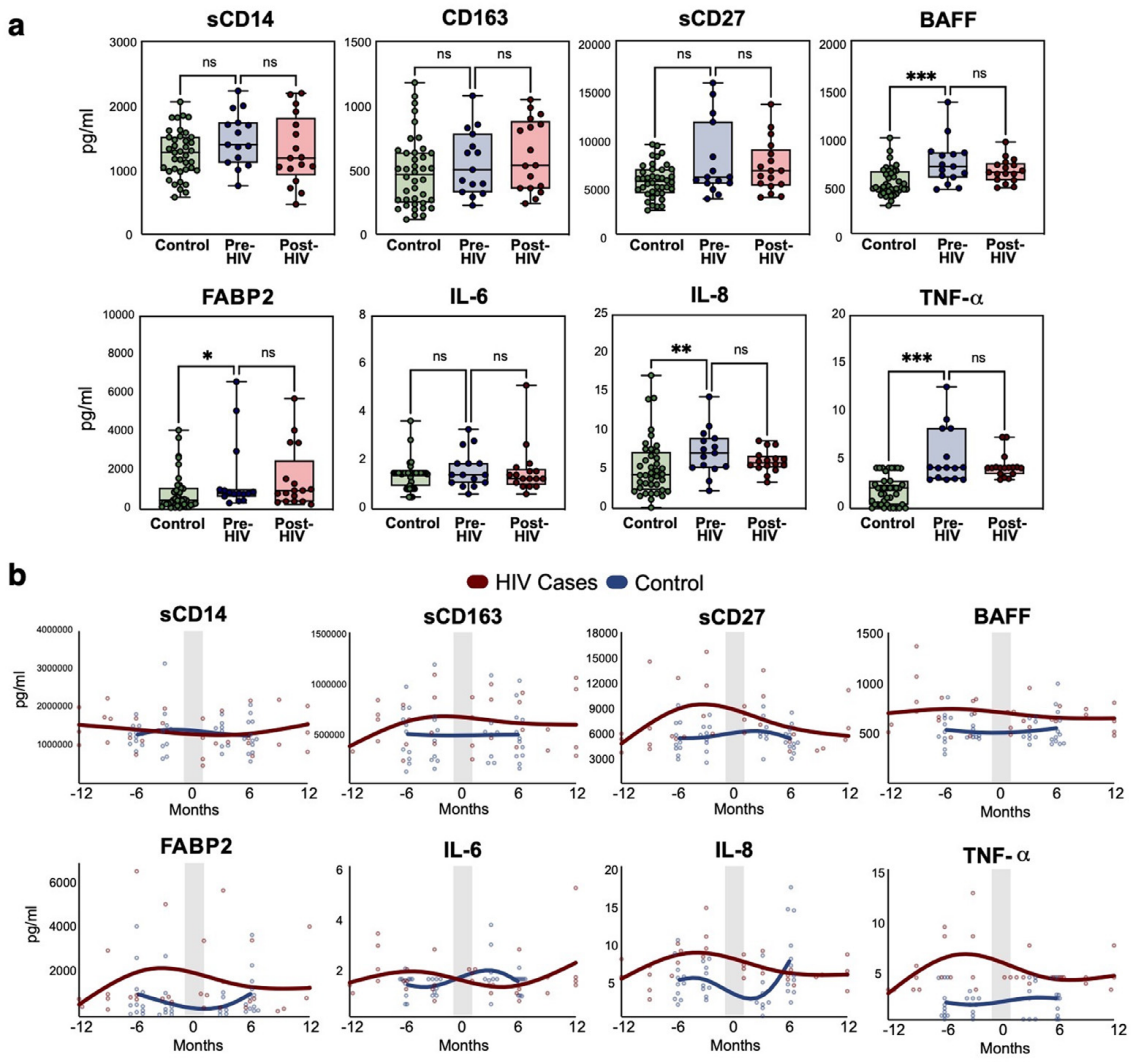
**Cytokines changes with HIV acquisition and seroconversion. (a)** Serum cytokines were quantified and compared between HIV cases (pre- and post-HIV) and control persons. *p*-values were calculated using Kruskal-Wallis tests with * <0.05, ** <0.01, and ***<0.001. Box line denotes median and whiskers min to max values. **(b)** Visualization of cytokines over time between HIV cases (red) and control persons (blue). The shaded area is the time of HIV seroconversion. Lines created using smoothing splines.(For interpretation of the references to color in this figure legend, the reader is referred to the web version of this article.)

Serum cytokines and immune biomarkers known to be important in HIV were also quantified. Prior to HIV acquisition, the HIV cases group showed higher B cell activating factor (BAFF) (*p*=<0.001), fatty acid binding protein (FABP2) (*p*=0.05), IL-8 (*p*=0.01), and TNF-α (*p*=<0.001) (Figure 5A). The control group showed little change in cytokine and immune biomarkers over time, whereas the HIV cases showed clear increases in sCD27, FABP2, IL-8, TNF-α at the visit prior to HIV infection (Figure 5B). There were no significant differences in cytokines or immune biomarkers before and during acute HIV.

### Networks involving secondary bile acid metabolism are altered after early HIV seroconversion

**Figure 6:**
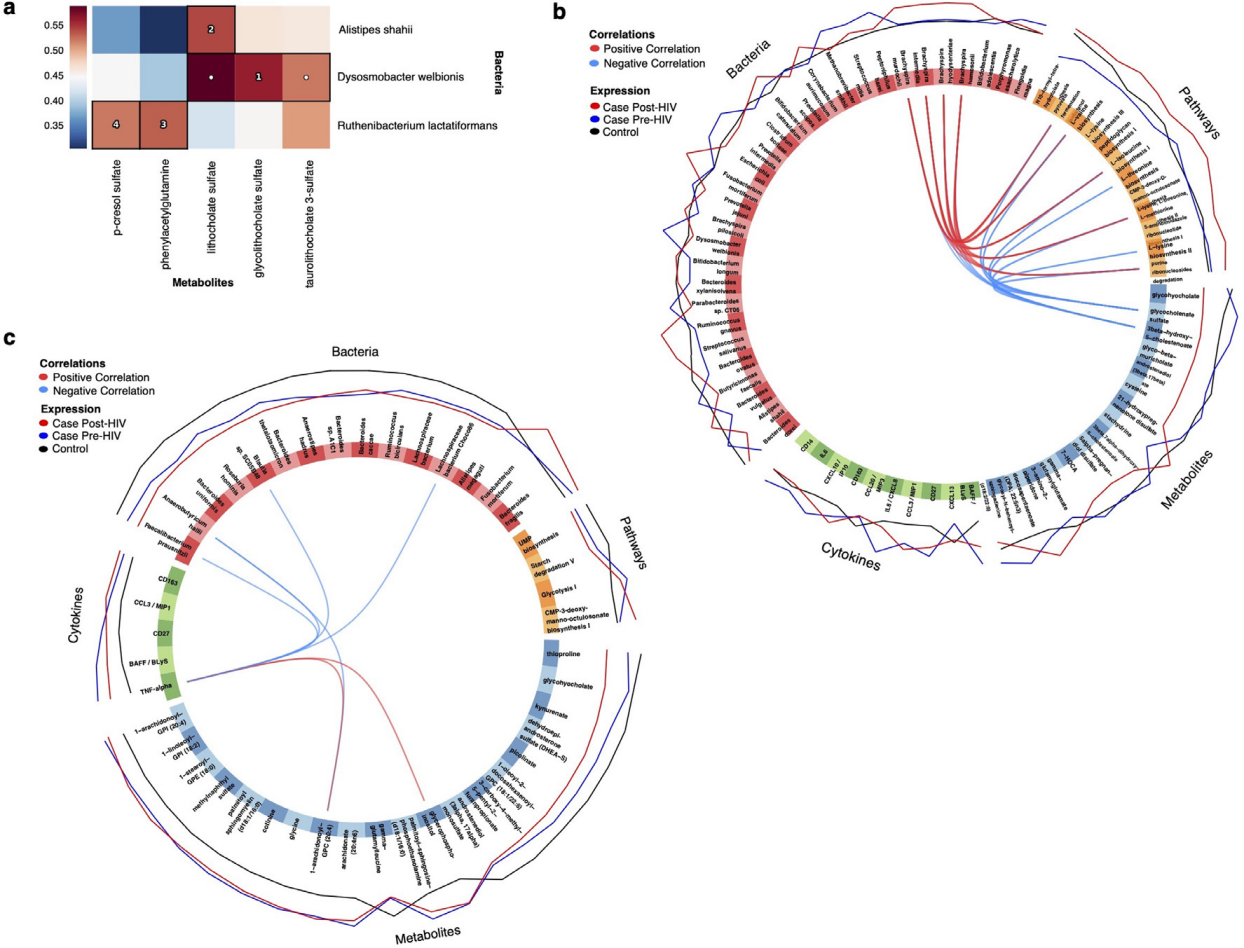
**Integrative multi-omic features associated with HIV acquisition and seroconversion. (a)** Hierarchical all against all association (HAllA) testing identified four clusters of bacteria and associated metabolites (identified by numbers 1 to 4). **(b and c)** Circos plots showing integrative multi-omics signatures associated with **(b)** HIV seroconversion (pre- and post-HIV cases) and **(c)** HIV acquisition (control and pre-HIV cases). Features are denoted on the circles, and lines connecting features within circles show positive (red) or negative (blue) associations. Lines on the outside show relative abundance of each feature between pre-HIV cases (blue), post-HIV cases (red), and control (black).(For interpretation of the references to color in this figure legend, the reader is referred to the web version of this article.)

To examine correlations between the microbiome and metabolome around HIV infection we used hierarchical all-against-all association testing (HAllA).^33^ Four densely associated clusters were identified between microbes and metabolites (Figure 6A**)**. The first cluster included *Dysosmobacter welbionis* and the secondary bile acids lithocholate sulfate and glycolithocholate sulfate; all of which decreased in abundance after early HIV seroconversion. Secondary bile acids, which are created by modifications from gut microbes, aid in nutrient absorption and have increasingly appreciated roles as signaling molecules.^34^ Cluster 2 included the secondary bile acid lithocholate sulfate and *Alistipes shahii*, which has been associated with liver disease and may be involved in bile acid metabolism.^35^ Both lithocholate sulfate and *Alistipes shahii* are decreased in abundance after early HIV seroconversion. Clusters 3 and 4 both included amino acid metabolites created by colonic bacteria amino acid metabolism (p-cresol sulfate and phenylacetylglutamine).^36^

### Integrative multi-omic analysis identified key features associated with HIV acquisition and early seroconversion

We used an N-integration approach within the ‘mixOmics’ framework to further identify novel multi-omic signatures related to HIV acquisition and seroconversion.^37^^,^^38^ Important relationships associated with early HIV seroconversion were identified along component 1 of the final model and included positive correlations between amino acid metabolism pathways and multiple *Brachyspira* species (Figure 6B). Though not identified in our prior modeling, an increase in *Brachyspira* during early HIV seroconversion can be appreciated by visualizing relative abundance over time (Figure 1E). Other important relationships included negative correlations between secondary bile acids (glycocholenate sulfate and 3beta-hydroxy-5-cholestenoate) and amino acid metabolism pathways, consistent with our prior findings using other approaches (Figures 4A and 6A). Important relationships associated with HIV acquisition were identified along component 2 and included positive correlations between TNF-α and bioactive lipids (glycerophosphoinositol and 1-arachidonol-GPC), and negative correlations between TNF-α and several bacteria known to have anti-inflammatory effects^39^^,^^40^ (Figure 6C). Both TNF-α and glycerophosphoinositol were identified as elevated in pre-HIV cases in prior analyses (Figures 4B and 5), suggesting these could be potential biomarkers for HIV acquisition risk.

## Discussion

Alterations in the gut microbiome have been repeatedly associated with chronic HIV infection, however the timing of the onset of dysbiosis remains unclear. This study sought to address this question by examining microbiome composition over time in individuals before and after early HIV seroconversion. We found limited changes in the microbiome during acute HIV (Figures 1 and 2), but pre-existing differences in microbiome composition were observed compared to control population (Figures 1 and 3). Longitudinal studies in non-human primates have repeatedly shown minimal changes in the gut bacterial composition following SIV infection,^41^^,^^42^ but until now similar studies had not been replicated in humans. Only one other longitudinal study in humans spanning the seroconversion period has been reported,^20^ and similarly found relatively few bacterial taxa alterations before and after HIV. These findings may seem in contrast to the existing literature on dysbiosis in HIV. Differences in study design (longitudinal vs cross-sectional), study population age, and the influence of confounders may explain some of this discrepancy.^17^ In addition, it is important to note that our observation period following HIV acquisition was too short to identify significant shifts in microbiome occurring during chronic HIV. Indeed, Rocafort et al examined longitudinal samples starting during acute HIV infection and found that HIV-specific microbiome changes were not present until chronic HIV stages.^43^ Finally, both our study and Chen et al found that many of the microbiome alterations previously associated with HIV, such as decreased *Bacteroides*, were in fact present prior to HIV acquisition. These findings provide evidence to shift our perception of gut dysbiosis from occurring as a result of HIV infection to potentially contributing to HIV infection.

Increased *Fusobacterium mortiferum* was observed during acute HIV acquisition in our study. Multiple prior studies have also found increased *Fusobacterium* spp associated with HIV infection. Fusobacterium are anerobic, butyric acid producing bacteria classically found in the oral cavity, and the presence of Fusobacterium in the gut has been associated with development of colorectal cancer and other inflammatory diseases.^44^ It is hypothesized that mucosal inflammation disrupts the gut anaerobic environment allowing for growth of aerotolerant bacteria, such as those which often reside in the oral cavity.^45^ Our data may support this as a potential mechanism triggering the development of dysbiosis later seen in chronic HIV.

While we did not observe large changes in the microbiome during acute HIV acquisition, we did identify pre-existing gut microbiome alterations that may increase susceptibility to HIV (Figure 3). The vaginal microbiome has been implicated in HIV susceptibility in women, with higher microbial diversity and non-*Lactobacillus* dominant communities associated with HIV acquisition in multiple studies.^46^^,^^47^ Until recently,^20^ similar associations had not been observed in the gut microbiome. We found increased *Megasphaera elsdenii* in persons who acquired HIV. Its role in the human gut is not yet known,^48^ but in the vaginal microbiome *Megasphaera elsdenii* prevalence is characteristic of bacterial vaginosis and has been associated with HIV risk in female sex workers.^47^^,^^49^ In that setting it induces dendritic cell activation and pro-inflammatory cytokine production,^50^ which could contribute to HIV susceptibility.

Decreased *Bacteroides* microbiome has been repeatedly associated with HIV infection,^51^ however our study suggests this depletion precedes HIV acquisition and is unique to those who acquired HIV suggesting a potential role in susceptibility. Importantly, similar findings were observed by Chen et al.,^20^ including several of the same species (*Bacteroides caccae, Bacteroides ovatus, Bacteroides uniformis*), using samples from the MACS which have notable differences in age, race/ethnicity, and collection era compared to our study. Multiple Bacteroides species, including *Bacteroides uniformis*, induce IL-10 and promote regulatory T cell accumulation,^52^ thus loss of these bacteria could contribute to a chronic inflammatory state which heightens HIV susceptibility. Other bacteria involved in regulating gut inflammation include *Ruminococcus* and *Akkermansia muciniphila*, both of which are decreased in chronic HIV.^15^^,^^53^^,^^54^ Our study did find decreases in these bacteria in the HIV cases group both prior to and during acute HIV (Supplementary Table 2); however, these did not meet statistical significance possibly due to the limited sample size and short follow-up period after HIV in our study.

While large changes in microbiome composition were not observed during acute HIV acquisition, we did identify alterations in several metabolites regulated by gut bacteria in early HIV infection. Metabolites in the secondary bile acid lithocholic acid pathway, which are created by microbial bile salt hydrolases, were decreased during acute HIV. In addition to aiding in nutrient absorption, bile acids also have function both in maintaining but barrier health as well as contributing to inflammation.^55^^,^^56^ Deviations in bile acid metabolism has been associated with HIV in other studies,^21^ and further investigation into the bile acid-microbiome interactions may identify novel pathways regulating mucosal inflammation in early HIV.

The role of sphingolipids in immune signaling and inflammation is an emerging area of interest. Sphingosine 1-phosphate and ceramide metabolism have been implicated in diseases such as inflammatory bowel disease, rheumatoid arthritis, and asthma.^57^ Sphingolipid signaling can be a mediator in TNF-α pathways, and indeed these markers were highly correlated in our multi-omics analysis suggesting this may be a key pathway related to HIV susceptibility (Figure 6C). Sphingolipids also have roles in membrane fusion and HIV-1 entry by facilitating CD4-CCR5-gp120 fusion complex formation,^58^ which may represent another mechanism mediating associations with HIV acquisition. Other biomarkers that may suggest susceptibility in our study included elevated soluble CD27, BLyS/BAFF, IL-8, TNF-α, and fatty acid binding protein (FABP2). Many of these cytokines contribute to either T cell activation (sCD27) or inflammation (IL-8, TNF-α). FABP2 can be a marker of gut mucosal inflammation, which is known to heighten HIV susceptibility.^59^^,^^60^ Lower BLyS/BAFF levels have been associated with relative protection from HIV in commercial sex workers.^61^ Validation of these lipid and immune biomarkers in larger studies is needed to guide further research. We did not find significant differences in cytokines within individuals before and during acute HIV, most likely due to the limited sample size and/or follow-up period prior to the onset of chronic HIV.

Limitations of this study include the relatively small sample size due to the limited availability of such unique longitudinal samples. Even with this limitation, many of our findings are highly consistent with another (larger) study.^20^ Based on sample availability, our study included only young men who have sex with men and thus generalizability beyond this population is limited. Additional studies including women and men who have sex with women will be important to better understand if these findings translate. Our study population included predominantly persons who use drugs. While this is a vulnerable and critical population to study, it may also limit generalizability. Our study population included predominantly racial and ethnic minorities which is of great importance given the disproportionate burden of HIV in these populations.^62^ The availability of detailed behavioral data is a great strength of our study. While we cannot completely mitigate unmeasured confounders or selection bias, our risk-behavior matching approach will help reduce this bias. As it is known that sexual practices and substance use can influence the microbiome in people living with HIV,^18^^,^^63^ we carefully matched our control group in terms of drug use and receptive anal intercourse partners to reduce influence of these confounders in our analyses. Despite these efforts, the HIV cases group did trend toward greater drug use which could indicate increased sexual risk behavior. Thus, we cannot fully exclude the influence of sexual behavior on the observed microbiome differences and HIV acquisition.

This study offers a rare view of the early effects of HIV acquisition on the gut microbiome and blood metabolic and immune profiles. Our data support a model in which pre-existing microbiome alterations contribute to HIV acquisition, with further dysbiosis developing over time in chronic HIV disease. Systemic inflammation, signified by elevated cytokines/biomarkers and bioactive lipids, may also contribute to susceptibility. Further studies to identify a systemic or microbiome “signature” of HIV susceptibility may help guide targeted prevention efforts.

## Contributors

JAF, FL, NHT, GMA conceived of and designed the study. JLC, BM, RD, MK, SS, PMG designed and provided study samples and data collection. SZ, JE performed experiments. JAF, FL performed data analysis. JAF, FL wrote the original manuscript draft and all authors contributed to review and editing the final manuscript. JAF and FL have directly accessed and verified the data reported.

## Data sharing statement

Sequence data from this study have been deposited in BioProject with the accession PRJNA836336. Metabolomics and cytokine data from this study are available in Dryad (https://doi.org/10.5061/dryad.np5hqbzx5).

## Declaration of interests

The authors have no conflicts of interest to declare.

## Appendix Supplementary materials

Image, application 1

Image, application 2

Image, application 3

Image, application 4

Image, application 5
