# Supplementary material for: Gut dysbiosis and inflammatory blood markers precede HIV with limited changes after early seroconversion
Source: eBioMedicine. 2022 Sep 27;84:104286. doi: 10.1016/j.ebiom.2022.104286 (PMC9520213; doi:10.1016/j.ebiom.2022.104286)
Supplement: Supplementary file 5 [file mmc5.docx]

**CAPTIONS FOR SUPPLEMENTARY MATERIAL**

Supplementary Table 1: Alpha diversity statistics for all samples

Supplementary Table 2: All results from Maaslin2 microbiome analysis

Supplementary Table 3: Random forests feature selection for microbiome data

Supplementary Table 4: Random forests feature selection for metabolomics data
